# 23 years of mTOR inhibition for tuberous sclerosis: Practical considerations

**DOI:** 10.1016/j.neurot.2026.e00959

**Published:** 2026-07-10

**Authors:** David Neal Franz

**Affiliations:** Department of Pediatrics and Neurology, University of Cincinnati College of Medicine, Cincinnati Children's Hospital Medical Center, Neurology, MLC 2015, 3333 Burnet Ave, Cincinnati, OH 45229–3039, USA

**Keywords:** mTOR, Giant cell astrocytoma, Tuberous sclerosis complex

The cause of tuberous sclerosis complex (TSC) is the hyperactivation of the protein kinase mTOR (mechanistic target of rapamycin) [1,2]. This occurs because of mutations in either the TSC1 gene (protein product hamartin) or the TSC2 gene (protein product tuberin). These proteins form a complex that act to moderate the activity of mTOR. These observations led to the use of mTOR inhibitors to treat various manifestations of tuberous sclerosis [2].

Approximately 30% of patients with TSC develop subependymal giant cell astrocytoma (SEGA's). SEGA's arise from subependymal nodules (SEN), which occur far more frequently in TSC patients with an incidence of 70–80%. Histologically, SEN's are indistinguishable from SEGA's. These lesions typically occur within the ventricular system, with SEGA's occurring primarily at the foramen of Monro [3]. However, they can develop at other locations. Astrocytic hamartomas present in the retina can also grow over time and represent a subtype of giant cell astrocytoma. Prior to the advent of routine screening for SEGA, as well as for other manifestations of TSC, e.g. renal angiomyolipoma, pulmonary lymphangioleiomyomatosis (LAM), these tumors often presented with signs and symptoms of increased intracranial pressure due to CSF obstruction.

Initially, the only treatment available for SEGA's was surgical resection. While this can be successful, it is important that a gross total resection be achieved, as any residual tumor will continue to grow and eventually produce symptoms. Our group was the first to employ mTOR inhibitors to treat giant cell astrocytomas in tuberous sclerosis, first rapamycin, and then, in an open label trial, everolimus [[2], [3], [4]]. These studies were remarkable in that all treated patients experienced reduction in tumor volume and avoided resective surgery. Since then, mTOR inhibitors rapamycin or everolimus have been evaluated in a variety of open label and randomized double-blind studies. These have resulted in regulatory approval for multiple manifestations of TSC: SEGA, renal angiomyolipoma, refractory epilepsy, LAM, facial angiofibroma, and cardiac rhabdomyomas, among others.

mTOR inhibitors in essence reverse the genetic defect present in tuberous sclerosis complex. While they might be started for a specific clinical concern, for example SEGA or renal angiomyolipoma, TSC patients receiving mTOR inhibitors experience benefits in every aspect of their disease. This would not be the case for surgical procedures, antiseizure medication, or other treatments. mTOR inhibitors have been repeatedly shown to be safe and well-tolerated [4,5]. However, side effect management is critical to maintaining compliance and continued benefit [6]. mTOR inhibitors have immunosuppressive properties and can adversely affect wound healing. However, rapamycin and everolimus bind avidly to mTOR. This allows treatment to be interrupted during times of illness or surgery without loss of efficacy.

In the April 2026 issue of Neurotherapeutics, Su et al. [7] describe their center's experience with the use of everolimus for the treatment of SEGA in patients with TSC. Their observations with everolimus confirm and amplify the findings of prior studies, albeit with some novel differences. For example, a lower burden of SEN's is positively associated with a greater reduction in lesion volume. While essentially all patients exhibit a reduction in SEGA volume, those with TSC1 mutations are more likely to do so. This finding is consistent with the general finding that patients with TSC1 mutations typically have a less severe phenotype then those with TSC2 mutations. The authors rightly point out the continuum between SEN's and SEGA's. In this study SEGA's were defined as lesions ranging in diameter from 0.5 to 1 cm or greater. This was based on a variety of consensus statements from the past. It is true that the larger subependymal nodules are more likely to be symptomatic as well as to become larger over time. Various criteria have been proposed for identifying when a subependymal nodule has become a giant cell astrocytoma: Contrast-enhancement on MRI, elevated lactate peak on magnetic resonance spectroscopy, ventricular obstruction, serial growth, among others. These criteria have various advantages and disadvantages. However, the most definitive indicator of an SEN being a SEGA is lesion growth on serial imaging studies. This is supported by existing literature and extensive clinical practice [[2], [3], [4], [5]]. Presymptomatic identification of a SEGA is the primary reason that serial neuroimaging studies are recommended in patients with TSC. The larger a SEGA happens to be when treatment is initiated, the more rapid and dramatic its response is likely to be. For example, in our initial open label trial of everolimus for SEGA, the patient with the greatest reduction in volume was the patient with the greatest lesion volume at treatment onset. This, despite the patient achieving a maximum everolimus serum concentration of only 2.8 ng/mL during the primary phase of the study [3]. mTOR inhibitors can also be used for acute treatment of symptomatic SEGA's in selected cases [8].

Currently, mTOR inhibitors are initiated for a wide variety of indications in TSC patients, rather than primarily for giant cell astrocytomas. Thus, some SEGA's certainly receive treatment before they produce symptoms or demonstrate progressive growth. mTOR inhibitors are not tumoricidal in patients with TSC. Rather, they change the biology of TSC associated lesions such as SEGA, causing a reduction in volume. After the initial response, tumor volume fluctuates around a new baseline which remains below their initial volume [5]. This does not reflect a treatment failure or lack of response. Rather, mTOR inhibitors produce a change in the biology of the hamartomas in a specific patient with TSC. As such they can be considered as a disease modifying therapy, analogous to TNF inhibitors for juvenile idiopathic arthritis for example [9].

The current accepted therapeutic range for mTOR inhibitors is based on their use as immunosuppressive agents in the setting of organ transplantation. As the paper by Su et al. [7} makes clear, this range is often excessive for specific patients with tuberous sclerosis. Specifically, serum concentrations in the low therapeutic range (or even in the subtherapeutic range) are typically sufficient to produce a clinical response. The strategy outlined in the current paper, namely using the lowest mTOR inhibitor dose that produces the desired clinical effect, represents the best clinical practice in this context. This strategy decreases the incidence and severity of adverse effects, and it is crucial for long-term tolerability of these medications [9].

Current research involving mTOR inhibitors and TSC involves their use earlier in life, with the view to preventing or ameliorating epileptic seizures, autism, and developmental delays. These are among the most disabling aspects of this condition. mTOR inhibitors are also used to treat hemodynamically significant cardiac rhabdomyomas prenatally, by administering these agents to the mother of an affected fetus [10]. Prenatal treatment with mTOR inhibitors represents the ultimate in preventative treatment for this condition, at least until, and if, specific genetic therapies are available.

## Author contributions

I am the sole author of this commentary.

## Declaration of competing interest

I have no conflicts of interest to declare.
